# Real-life results of sofosbuvir based therapy in chronic hepatitis C -naïve and -experienced patients in Egypt

**DOI:** 10.1371/journal.pone.0184654

**Published:** 2017-10-05

**Authors:** Ahmed Nagaty, Ekram W. Abd El-Wahab

**Affiliations:** 1 Consultant of Hepatology and Infectious Diseases, Ministry of Health, Alexandria, Egypt; 2 Tropical Health Department, High Institute of Public Health, Alexandria University, Alexandria, Egypt; Nihon University School of Medicine, JAPAN

## Abstract

**Background:**

More than ten million Egyptians are infected with HCV. Every one of them is going to infect about three to four persons every year. Treating those patients is a matter of national security. A dramatic improvement in hepatitis C virus (HCV) infection treatment was achieved in the last five years. A new era of direct-acting antivirals is now dawning in Egypt.

**Objective(s):**

We share in this report our clinical experience in treating chronic HCV Egyptian patients with Sofosbuvir based regimens to evaluate its safety and efficacy on real life practical ground.

**Methods:**

A total of 205 chronic HCV patients (195 naive and 15 experienced) were enrolled in the study. Patient were treated with Sofosbuvir+Ribavirin 24 weeks as standard of care. Two interferon eligible patients were treated with PEG-INF+ Sofosbuvir+Ribavirin for 12 weeks. The primary efficacy endpoint was the proportion of patients with sustained virologic response at 24 weeks after cessation of therapy.

**Results:**

The overall response rate was 97.1%. Sustained virological response rate did not differ among treatment-naive patients and patients with previous history of IFN-based therapy. Portal hypertension, prediabetes, and lack of early virologic response were predictors of non response. No clinically significant treatment-emergent adverse effects were noted. No treatment discontinuation was encountered.

**Conclusion:**

In the real-life setting, Sofosbuvir based regimens for 24 weeks has established an efficacious and well tolerated treatment in naïve and experienced patients with chronic HCV genotype 4 infection; although shorter treatment durations may be possible. However, patient follow up should extent to at least 6 months post-treatment and verifying viral load on yearly basis is warranted to track any late relapse.

## Introduction

A revolution in hepatitis C virus (HCV) infection treatment was achieved in the last seven years marked by the introduction of the direct-acting antivirals (DAAs). Sofosbuvir is a DAA and the backbone of oral anti HCV therapy. It is a uridine nucleotide analogue that selectively inhibits HCV NS5B polymerase and has potent activity, pangenotypic coverage (G1-G6) and high barrier to resistance [1–4]. Egypt has the highest HCV prevalence worldwide estimated nationally at 4.1% [5]. HCV genotype 4 is also the predominating strain in Egypt as has been revealed in more than 90% of the infections [6–9].

The National Control Strategy for Viral Hepatitis was first developed in 2008 and aimed to treat 20% of patients by the year 2012 under subsidized schemes. Treatment was based on pegylated interferon (peg-INF) in combination with ribavirin (RBV). However, advanced liver care was not financially feasible and only patients with relatively higher chances of cure had access to treatment. Also, children were excluded from this strategy [10]. Moreover, this regimen had modest success rates (40–70% for genotype 4), poor tolerability and difficult administration [7, 11–13]. New regimens involving direct-acting antiviral agents (DAAs) have recently been approved for the treatment of genotype 4 HCV. These regimens appear to offer improved rates of sustained virological response (SVR) in treatment-naive and previously treated patients infected with genotype 4 HCV. A significant pool of patients has received these regimens and results concerning efficacy and safety are promising. The efficacy of SOF-based treatment regimen has been evaluated in phase II and phase III trials demonstrating SVR rate reaching 96% [3].

In 2014, Egypt stepped up its efforts and started a new campaign for HCV treatment [10] aimed at eliminating the large reservoir of infection thus preventing the spread of the disease which is still ongoing through iatrogenic sources due to laxity in infection control measures [14, 15]. The goal is to reach <1% infection rate by 2030. The new strategy has received support from several partners including the WHO, Pasteur’s Institute and the Centers for Disease Prevention and Control. The national program employs web-based online patient registration and scheduling appointments (>1.8 million so far) to be enrolled for DAAs treatment at the affiliated HCV treatment centers found all over the country. More than 900,000 Egyptians have been treated for hepatitis C since January 2016 using the DAAs that have a proven efficacy in tackling the disease [10, 16, 17].

The introduction of the new DAAs marked the beginning of a new era in HCV therapy in Egypt. Nevertheless, real-life results concerning the efficacy and safety of this therapy for HCV genotype 4 in Egypt are still scarce. In fact, efficacy and safety rates reported in randomized control trial can be lower in community-based practice settings since concomitant diseases or constitutional factors may yield additional aspects and knowledge valuable for the future management of affected patients [18]. The present work aimed therefore to assess the clinical effectiveness of sofosbuvir based therapy in chronic hepatitis C -naïve and -experienced Egyptian patients predominately infected with genotype 4 [6] and to identify the possible predictors of non-response to treatment.

## Methods

### Compliance with ethical standards

The study was approved by the institutional review board and the ethics committee of the High Institute of Public Health affiliated to Alexandria University, Egypt. The research was conducted in accordance with the ethical guidelines of the Declaration of Helsinki (2013) and the International Conference on Harmonization Guidelines for Good Clinical Practice. Informed consent was obtained from all individual participants enrolled after explaining the aim and concerns of the study. Data sheets were coded to ensure anonymity and confidentiality of patient’s data.

### Study design and patients

A prospective real life cohort study was conducted between June 2015 and May 2016 at a non-governmental Liver Center in Alexandria (Egypt). This center offers treatment free of charge for patients not covered by health insurance. The present work represents an observational study in which sofosbuvir was administered to all participants as standard of care treatment. The anti-HCV drugs were offered by Pharco Pharmaceutical company in Alexandria. The study patients comprised chronic HCV positive patients (naïve and -experienced) eligible for receiving DAAs. The criteria for inclusion were: ages above 18 years, both sex, HCV-RNA positive PCR, complete blood picture within normal ranges and all eligible cases for treatment according to international guidelines (Child A, Child B). Those found ineligible for the treatment were excluded from the study [Age under 18, history of malignancy, untreated thyroid disease, active substance abuse, and hepatic decompensation [(Child C, Child D), (TSB >3mg/dl, Albumin<2.8 g/dl, INR>1.3, platelets <50000/cmm)], presence of ascites on Ultra sound, uncontrolled diabetes (HbA1C >9%), current or planned pregnancy, end stage renal disease or serum creatinine greater than 1.5 mg/ml, continued pattern of alcohol abuse >40g/day in the last 6 month, WBC <3000/cmm, Hemoglobin <10g/dl, hepatocellular carcinoma (HCC), retinopathy, HIV, and obese patients (Body Mass Index (BMI)>35)]. Difficult to treat patients included those having FIB-4 >3.25, albumin ≤3.5, total Bilirubin >1.2 mg/dl, INR >1.2 and platelet count <150000 mm3 [19, 20].

### Clinical and laboratory investigations

All enrolled patients were interviewed using structured predesigned questionnaire to collect sociodemographic data and were subjected to complete medical examination (general and abdominal) including the estimation of the BMI. Baseline laboratory investigations for proper selection of eligible cases for treatment comprised: complete Blood Count (CBC), fasting blood glucose, liver function tests (total serum bilirubin (TSB), ALT, AST, prothrombin time, prothrombin activity, international normalized ratio (INR), serum albumin), serum creatinine, Anti Nuclear Antibody (ANA), Thyroid Stimulating Hormone (TSH), free T3, free T4, alpha fetoprotein (AFP), antibilharzial Ab, HIV Ab, HBsAg as a marker for HBV infection [Enzyme-linked immunosorbent assay (3^rd^ generation ELISA kit DIALAB^®^, Austria), HCV RNA Quantitation by Real-Time PCR using Cobas Ampli Prep/Cobas TaqMan HCV-RNA assay (Roche Diagnostics; Pleasanton, CA, USA) with a threshold of detection = 15 IU/ml, Anti-bilharzial Ab: [Indirect hemagglutination test (IHA) (Fumouze Diagnostics, France)]. Pregnancy test was done for women in child bearing age. ECG and fundus examination were done for all patients. One patient was found HbsAg positive but was enrolled in the study. Those found seropositive for bilharziasis received antibilharzial treatment regimen for 10 days prior to enrollment in the HCV treatment cohort.

All patients underwent abdominal ultrasound examination to check for the general condition of the liver and other abdominal organs. The FIB-4 score was calculated using Sterling’s formula: Age (years) X AST[IU/L]/PLT[10^9^/L] X ALT[IU/L] [21].

### Regimen of combination therapy

Since genotype 4 is predominating in Egypt [6–9], all enrolled patients received sofosbuvir/ribavirin combination therapy for 24 weeks. Two young patients were counseled to receive treatment for 12 weeks only, and as they were young age and eligible for interferon therapy, they received sofosbuvir/pegyinterferon/ribavirin combination therapy. Sofosbuvir was given at 400 mg oral daily dose. Ribavirin (Rebetol; Schering-Plough K.K.), was given in two divided daily oral doses adjusted to body weight (800 mg for weights < 50 kg, 1,000 mg for weights 50–65 kg, 1,200 mg for weight 65–80 kg, and 1,400 mg for weights >80 kg). Peg-IFN-α-2b (Pegintron; Schering-Plough K.K., Kenilworth, NJ) was given in weekly doses adjusted to body weight according to the manufacturer’s instructions at 1.5 μg/kg/week. One patient was found to have HCV/HBV co-infection with evidence of active viral replication. This patient received the standard of care for HCV plus entecavir at 1 mg PO qDay (as the patient was defaulter to lamivudine before enrollment for receiving HCV therapy) that will be continued for life with regular monitoring of HCV and HBV viral load and liver enzymes throughout the course of treatment and after achieving SVR. To compensate for Hb drop attributed to Ribavirin, folic acid and iron supplements were given to patients. All patients found positive for Anti-shistosomal Ab received praziquantal 60 mg/kg /day orally in 3 divided doses for 10 day before starting DAAs.

### Follow up

Patients underwent follow up during the 24 weeks of treatment period and for 24 weeks after treatment. Laboratory investigations, including CBC, liver function and renal function tests were repeated at 4, 8, 12, 16, 20 and 24, 36, and 48 weeks during and post-treatment. At week 4, 12, 24 of treatment and at 12, 24 weeks post-treatment all enrolled patients were subjected to qRT-PCR for viral load to detect early (EVR) and sustained (SVR) virologic responses. Early virologic response was defined as undetectable HCV-RNA or below the lower limit of quantification at 4 weeks of initiation of treatment, while the SVR24 was defined as undetectable HCV-RNA or below the lower limit of quantification at 24 weeks after the last dose of treatment (or at 48 week of starting treatment) as endpoint.

### Statistical analysis

Data were collected, revised, coded and fed to statistical software SPSS (Statistical Package for the Social Sciences) Version16.0 (SPSS, Chicago, IL, USA). Numeric data were analyzed using percentages, mean, and student t-test. For categorical data, univariate binary logistic regression analysis was done for all independent variables. Multiple logistic regression analysis with the forward stepwise variable selection was used to identify the independent predictors impacting response to treatment. All statistical analysis was done using two tailed tests and alpha error of 0.05. A *p* value <0.05 was set a level of significance.

## Results

### Patient demographics

**Fig 1:**
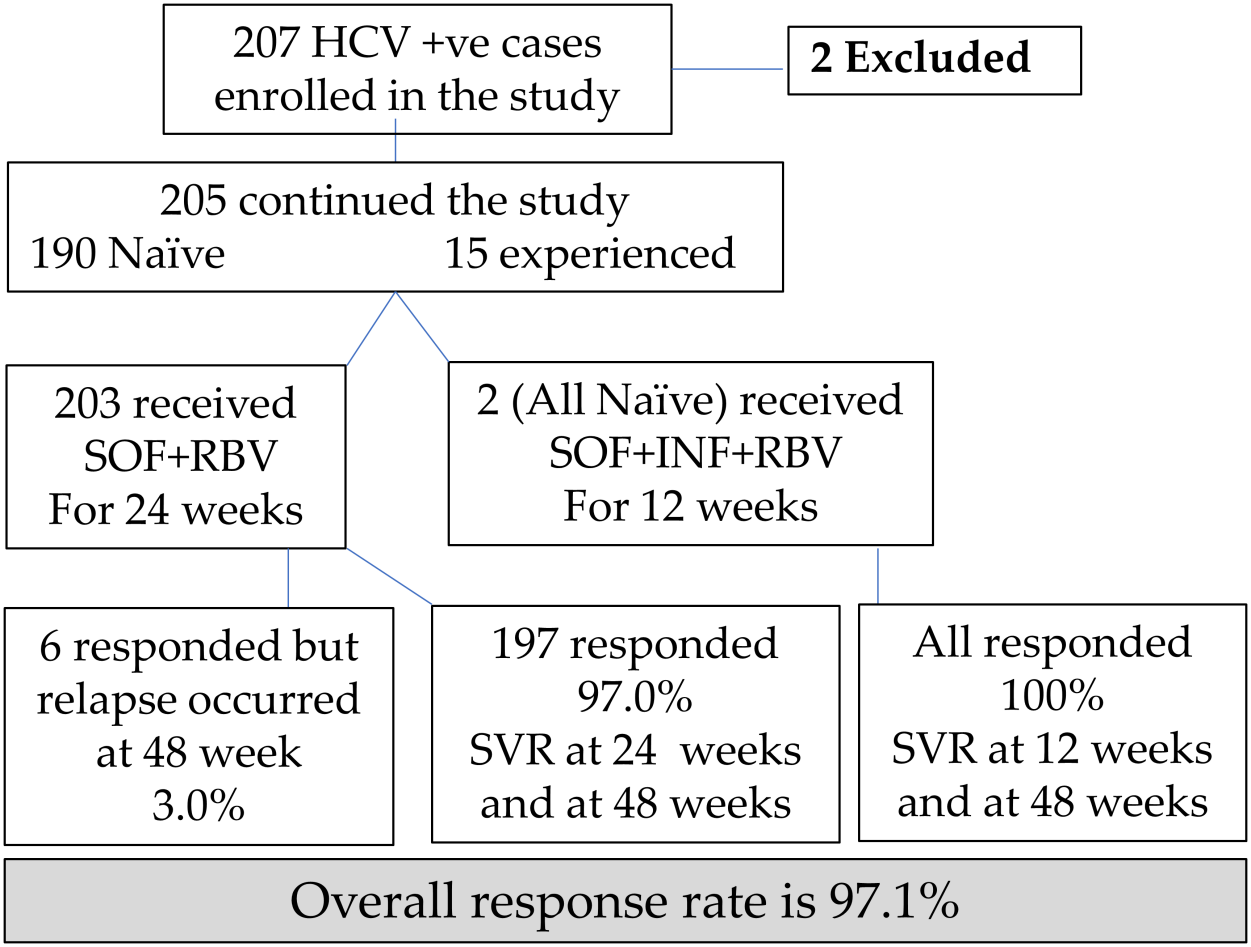
Patient disposition throughout the study.

**Fig 2:**
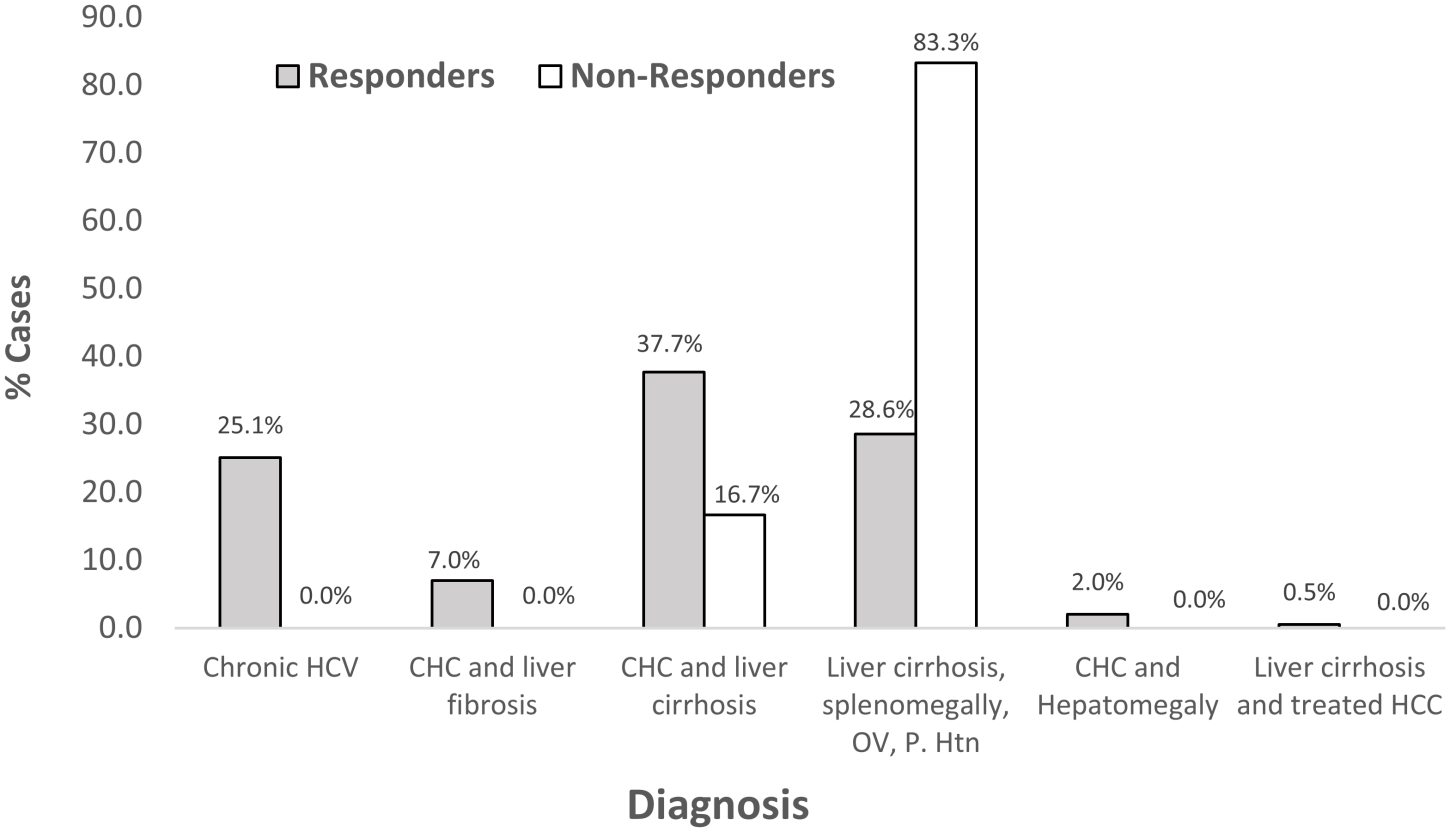
Patient diagnosis before enrollment.

**Fig 3:**
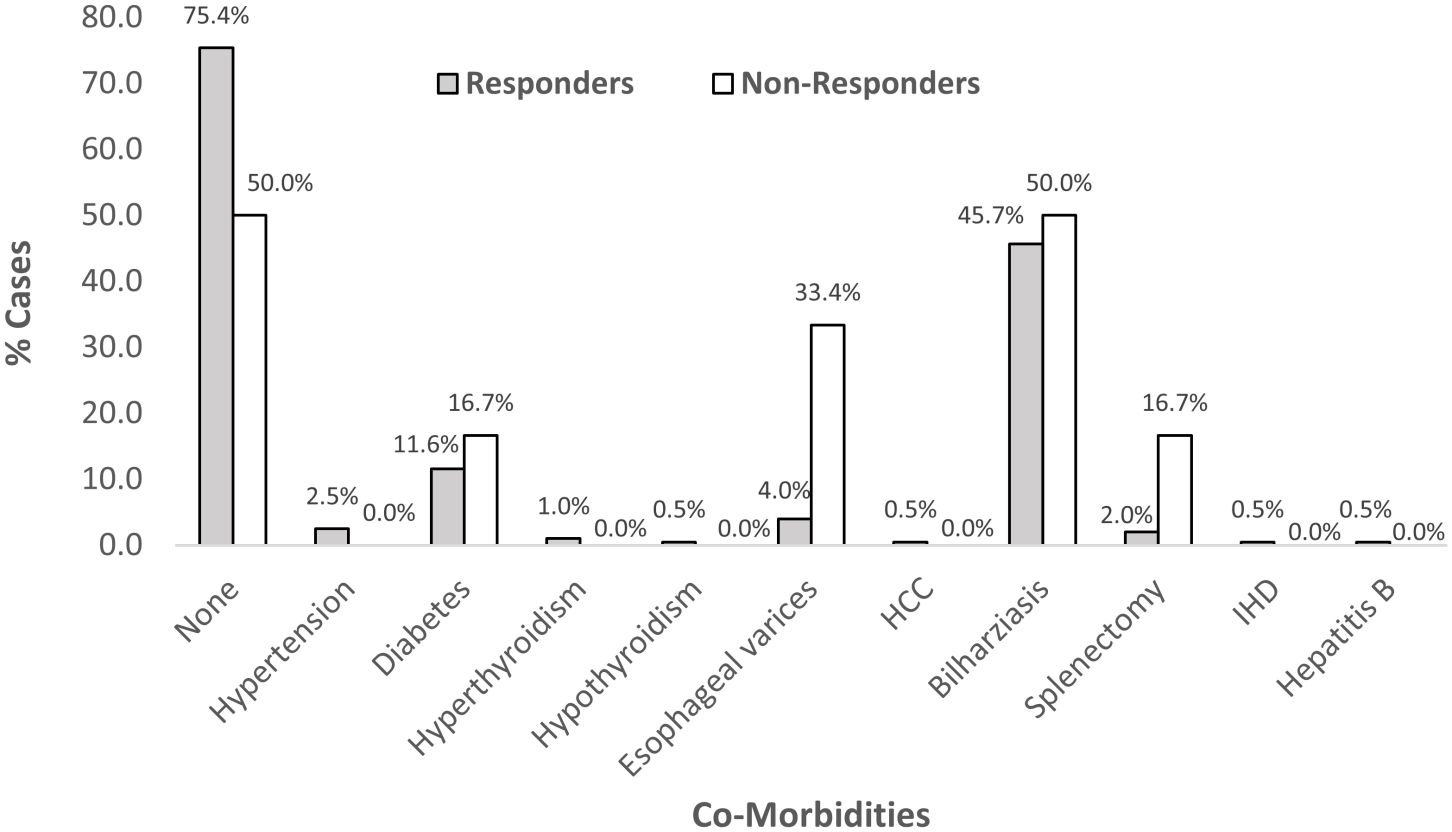
Co-morbidities among responders and non responders.

We enrolled 207 HCV +ve patients. Two patients denied having decompensated liver condition, received diuretics to mask ascites and deceive the investigator and thus be eligible for treatment. They were enrolled in the study cohort but developed hepatic encephalopathy after two weeks of starting treatment. They discontinued treatment and were excluded from the study cohort. About 205 patients continued the study and were followed for 24 weeks after treatment completion (Fig 1). Females were more presented than males (53.7% each). Their ages ranged between 20 and 85 years (Mean±SD = 49.6±11.5). The majority (80.5%) were rural residents. More than half of the patients (56.1%) were obese and most of them were non smokers (92.2%). At base line, 7.3% of all patients were treatment experienced, 11.1% were diabetics, 66.8% had cirrhosis, 11.1% had portal hypertension, 2.4% had splenectomy, one patient (0.5%) was HbsAg positive, and 1.5% were classified as difficult to treat. The majority of non responder were diagnosed as having liver cirrhosis, portal hypertension and esophageal varices (Fig 2). Co-morbidities were more frequent among non responders (50.0% *vs* 25.0% for responders) (Fig 3).

### Treatment response

**Fig 4:**
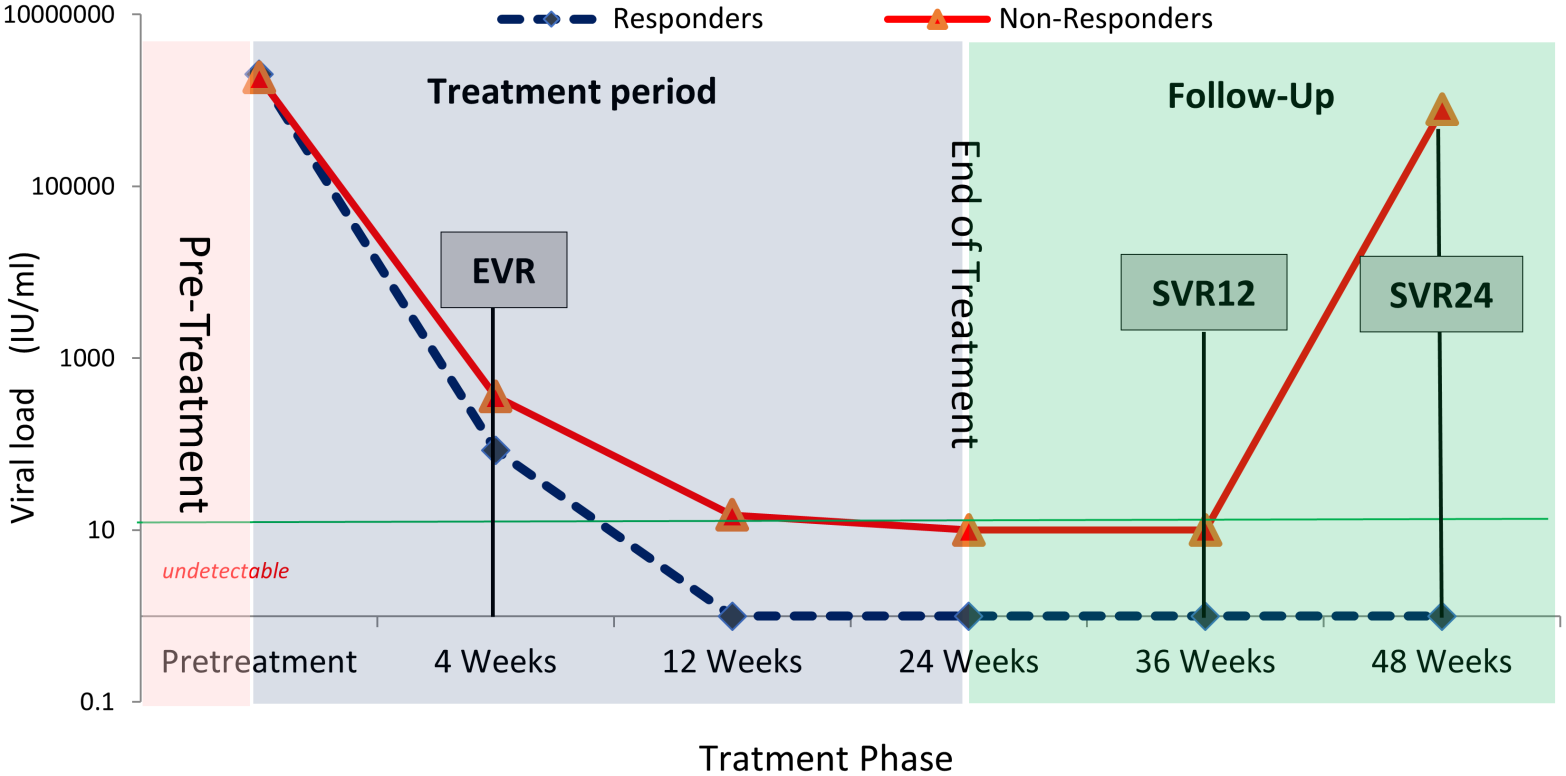
Virologic response during and after treatment periods.

By week four of treatment, all but 4 patients (98.1%) had cleared HCV RNA thus achieved EVR and all maintained virological suppression while receiving therapy (Fig 4). All patients (100.0%) achieved viral clearance as a treatment endpoint. However, after completion of treatment the relapse rate at 48 week of follow up was 3.0%, thus the SVR was achieved by 97.1%. The SVR12 was 100.0% and SVR24 was 97.1%. The response rate among treatment experienced patients was 100.0%. The majority of the treated patients experienced side effects in the form of fatigue, skin rash, drug eruptions, GIT disturbance and drop in Hb level. These were self-limited and were symptomatically managed, and none forced on therapy patients to discontinue the treatment.

**Fig 5:**
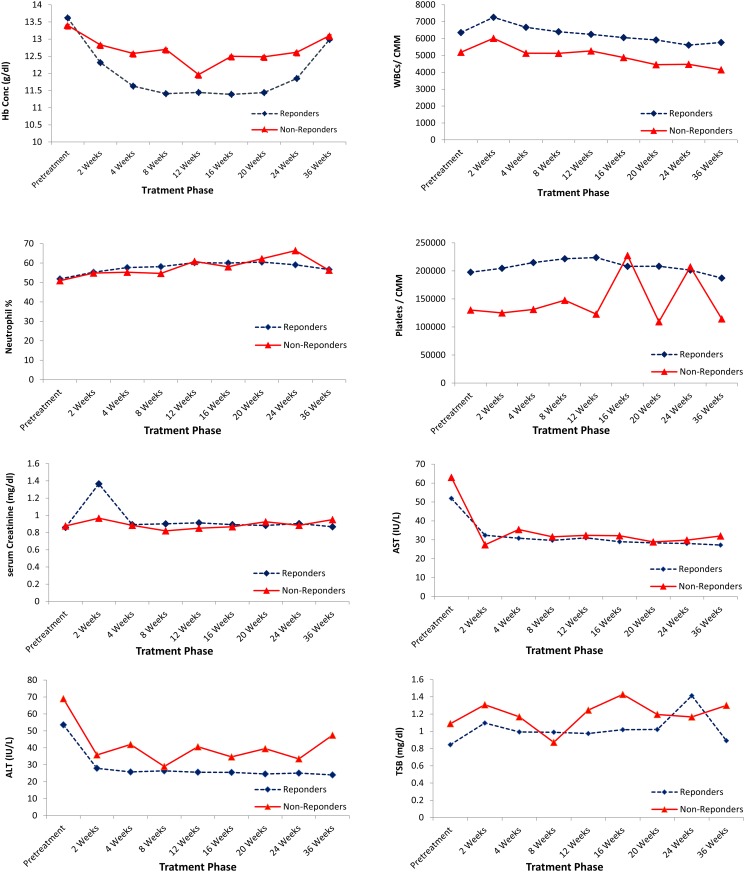
Hematological and biochemical changes over the course of treatment and during follow up.

SOF based therapy was well tolerated and did not affect significantly the blood picture. There was no significant difference between responders and non-responders regarding the effect of treatment on Hb level, WBCs count, neutrophil % or platelet count. The treatment resulted in decrease of liver enzyme level but slight increase in serum creatinine and TSB although levels were still within normal range (Fig 5).

In univariate logistic regression analysis, several factors were found significantly associated with non-response to HCV therapy (Tables 1 and 2). SVR24 was not achieved in those not achieving EVR [OR (95% CI) = 198 (15.7–2900), *p*<0.0001], having portal hypertension [OR (95% CI) = 19 (3.3–110.4), *p*<0.0001], splenectomy [OR (95% CI) = 9.8(1–103.7), *p* = 0.05], elevated AFP [OR (95% = 9.1(1.6–51.3) *p* = 0.01], high serum bilirubin [OR (95% CI) = 9.7(1.71–55.2) *p* = 0.01], low hemoglobin level [OR (95% CI) = 21(1.3–335.3) *p* = 0.03], prediabetic state [OR (95% CI) = 6.5 (1.1–39.1), *p* = 0.04], TFT-T3 [OR (95% CI) = 0.3 (0.2–1), *p* = 0.04], or TFT-T4 [OR (95% CI) = 1.2 (1–1.4), *p* = 0.046], and low platelet count [OR (95% CI) = 1 (1–1) *p* = 0.043]. In our multivariate stepwise logistic regression model, only prediabetics [OR (95% CI) = 38.2 (2.1–686.7). *p* = 0.013], not achieving EVR [OR (95% CI) = 736.7 (16.8–3200), *p*<0.001], and having portal hypertension [OR (95% CI) = 31.2 (1.9–501.7), *p*<0.015] appeared among factors impacting treatment response in the study cohort (Table 3).

**Table 1:** Patient demographics and baseline characteristics among responders and non-responders.

Variable	Total		SVR (n = 205)				OR [95% CI (LL-UL)]	*P*
			Responders		Non-Responders			
	no.	%	(n = 199)		(n = 6)			
			no.	%	no.	%		
**Age (years)**								
20-	12	5.9	12	100	0	0	ref	0.9
30-	48	23.4	47	97.9	1	2.1	1 [ND]	1.0
45-	107	52.2	102	95.3	5	4.7	3.4x10^7^ [ND]	0.99
60–85	38	18.5	38	100	0	0	7.9x10^7^ [ND]	0.99
**Mean±SD**	**Mean±SD = 49.6±11.5 Median = 50 [Range = 65 (20–85], Q1 = 41, Q3 = 58**		**49.3±11.6**		**50.6±3.6**		t = -0.8 *p* = 0.45	
**Gender**								
Male	95	46.3	90	94.7	5	5.3	6.05 [0.7–55.8]	0.1
Female	110	53.7	109	99.1	1	0.9		
**Residence**								
Urban	40	19.5	38	95	2	5.0		0.39
Rural	165	80.5	161	97.6	4	2.4	0.47 [0.08–2.7]	
**Smoking**								
No	189	92.2	183	96.8	6	3.2	ND	1.0
Yes	16	7.8	16	100	0	0.0		
**BMI**								
18- (Normal weight)	34	16.6	33	97.1	1	2.9		0.83
25- (Over weight)	56	27.3	55	98.2	1	1.8	0.6 [0.03–9.9]	0.72
30+ (Obese)	115	56.1	111	96.5	4	3.5	1.2 [0.1–11.0]	0.87
**Previous Treatment**								
Experienced	15	7.3	15	100	0	0.0		1.0
Naïve	190	92.7	184	96.8	6	3.2	5.2x10^7^ [ND]	
**Comorbidities**								
Yes	52	25.4	49	94.2	3	5.8	3.1 [0.6–15.6]	0.18
No	153	74.6	150	98	3	2.0		
**HBsAg**								
Negative	204	99.5	198	97.1	6	2.9	ND	1.0
Positive	1	0.5	1	100	0	0.0		
**Cirrhosis**								
Yes	137	66.8	131	95.6	6	4.4	7.3x107 [ND]	0.99
No	68	33.2	68	32.2	0	0.0		
**Splenectomy**								
No	200	97.6	195	97.5	5	2.5		
Yes	5	2.4	4	80.0	1	20.0	**9.8 [1–103.7]**	**0.05**
**Portal Hypertension**								
No	182	88.9	180	98.9	2	1.1		
Yes	23	11.1	19	82.6	4	17.4	**19 [3.3–110.4]**	**<0.0001**
**Difficult to treat**								
No	202	98.5	196	97	6	3.0	ND	1.0
Yes	3	1.5	3	100	0	0.0		
**Treatment Regimen**								
SOF+RBV	203	99	197	97	6	3.0	4.9x10^7^ [ND]	1.0
SOF+RBV+INF	2	1	2	100	0	0.0		
**EVR**								
Yes	201	98	198	98.5	3	1.5	**0.005 [0–0.064]**	**<0.0001**
No	4	2	1	25	3	75.0		

**Table 2:** Viral and host characteristics associated with virologic response among responders and non-responders.

		Total		SVR (n = 205)				OR [95% CI (LL-UL)]	*P*
				Responders		Non-Responders			
		no.	%	(n = 199)		(n = 6)			
				no.	%	no.	%		
**Viral load (IU/ml)**	Very low (<10.000)	7	3.4	7	100.0	0	0.0	ref	0.8
	Low (<100.000)	29	14.1	29	100.0	0	0.0	1 [ND]	1
	Intermediate (<1000.000)	88	42.9	86	97.7	2	2.3	3.7x10^7^ [ND]	0.99
	High (>1000.000)	81	39.5	77	95.1	4	4.9	8.4x10^7^ [ND]	0.99
**Anti-Bilharzial Ab**	Negative	111	54.1	108	97.3	3	2.7		0.83
	Positive	94	45.9	91	96.8	3	3.2	1.2 [0.23–6.02]	
**AFP**	Normal (up to 10 ng/ml)	166	81.0	163	98.2	3	1.8		**0.013**
	Elevated (>10 ng/ml)	39	19.0	35	89.7	4	10.3	**9.1 [1.6–51.3]**	
**TSH**	Below normal	10	4.9	10	100.0	0	0.0		0.7
	Normal (0.35–5 uIU/mL)	191	93.2	185	96.9	6	3.1	0.9 [0.5–1.8]	
	Elevated	4	2.0	4	100.0	0	0.0		
**T3**	Below normal	54	26.3	50	92.6	4	7.4	0.3 [0.2–1]	**0.04**
	Normal (1.68–3.5 pg/ml)	142	69.3	140	98.6	2	1.4		
	Elevated	9	4.4	9	100.0	0	0.0		
**T4**	Below normal	2	1.0	2	100.0	0	0.0	**1.2 [1–1.4]**	**0.046**
	Normal (0.9–1.9 ug/dL)	201	98	195	97.0	6	3.0		
	Elevated	2	1.0	2	100.0	0	0.0		
**ANA**	Normal (<1.0 IU)	190	92.7	185	97.4	5	2.6		0.39
	Elevated (>1.0 IU)	15	7.3	14	93.3	1	6.7	2.6 [0.3–24.2]	
**Prothrombin Time**	Normal (9.5–19.8 sec)	205	100	199	97.1	6	2.9	1.4 [0.76–2.6]	0.26
**PA**	Below normal (<80)	33	16.1	31	93.9	2	6.1	2.7 [0.5–15.4]	0.26
	Normal (80–100)	172	93.9	168	97.7	4	2.3		
**INR**	Normal (0.8–1.2)	170	82.9	166	97.6	4	2.4		0.3
	Elevated (>1.2)	35	17.1	33	94.3	2	5.7	2.5 [0.4–14.3]	
**Total Bilirubin**	Normal (up to 1 mg/dl)	167	81.5	165	98.8	2	1.2		**0.01**
	Elevated (>1 mg/dl)	38	18.5	34	89.5	4	10.5	**9.7 [1.71–55.2]**	
**AST**	Normal (up to 37 U/L)	78	38.0	78	100.0	0	0.0		0.99
	Elevated (> 37–198 U/L)	127	62.0	121	95.3	6	100.0	8x107 [ND]	
**ALT**	Normal (up to 40 U/L)	94	45.9	93	98.9	1	1.1		0.18
	Elevated (>40–260 U/L)	111	54.1	106	95.5	5	4.5	4.4 [0.5–38.2]	
**Serum Albumin**	Below normal (<3.5 g/dl)	20	9.8	20	100.0	0	0.0	0.65 [0.13–3.2]	0.6
	Normal (3.5–5.5 g/dl)	185	90.2	179	96.8	6	3.2		
**HBA1C**	Normal (up to 6.0)	154	75.1	149	96.8	5	3.2	1.2 [0.5–2.5]	0.68
	Elevated (> 6.0)	51	24.9	50	98.0	1	2.0		
**FBS**	Normal (70–110 mg/dl)	174	84.9	170	97.7	4	2.3	ref	0.12
	Prediabetics (111–125 mg/dl)	15	7.3	13	86.7	2	13.3	**6.5 [1.1–39.1]**	**0.04**
	Elevated (>126 mg/dl)	16	7.8	16	100.0	0	0.0	ND	0.99
**Serum Creatinine**	Normal (0.5–1.2 mg/dl)	199	97.1	193	97.0	6	3.0	1.7 [0.02–148.8]	0.8
	Elevated (>1.2 mg/dl)	6	2.9	6	100.0	0	0.0		
**WBCs**	Below normal (2.9x10^3^/cmm)	19	9.3	19	100.0	0	0.0	0.99 [0.9–1]	0.07
	Normal (4–11.7 x10^3^/cmm)	186	90.7	180	96.8	6	3.2		
**Neutrophil**	Normal	174	84.9	169	97.1	5	2.9		0.95
	Below normal	31	15.1	30	96.8	1	3.2	0.9 [0.04–21.6]	
**Platelet count**	Below normal (57 x10^3^ -)	62	30.2	58	93.5	4	6.5	1 [1–1]	**0.043**
	Normal (150 x10^3^-465 x10^3^/cmm)	143	69.8	141	98.6	2	1.4		
**Hemoglobin level**	Below normal	63	30.7	59	93.7	4	6.3	**21 [1.3–335.3]**	**0.03**
	Normal	142	69.3	140	98.6	2	1.4		
**FIB4**	F0-F1 (<1.45)	79	38.5	78	98.7	1	1.3	ref	0.23
	F2-<F3 (1.45–3.25)	84	41.0	82	97.6	2	2.4	1.6 [0.17–21.4]	0.6
	>F3 (>3.25)	42	20.5	39	92.9	3	7.1	6 [0.6–59.6]	0.13

**Table 3:** Multivariate stepwise logistic regression model for predictors of non response to anti HCV treatment.

	Predictors	B	S.E.	Sig.	Exp(B)	95.0% C.I. for EXP(B)	
**Step 10**	Prediabetics (FBS = 111–125 mg/dl)	3.64	1.5	0.013	38.2	2.1	686.7
	EVR	6.60	1.9	0.001	736.7	16.8	3200
	Portal Hypertension	3.44	1.4	0.015	31.2	1.9	501.7
	Constant	-6.5	1.5	0.000	0.0		

## Discussion

The current study cohort treated with SOF-based regimens attained rates of SVR at week 12 (100.0%) and at week 24 (97.1%) higher than those demonstrated in phase 3 clinical trials (82% for genotype 4) [2, 3, 22–26], and real-life study data [27]. In the later study, Elsharkawy and co-worker evaluated post-treatment data on two large study cohorts enrolled in National Treatment Programme for Hepatitis C in Egypt and treated with triple and dual sofosbuvir based regimens for 3 and 6 months respectively and followed up for 12 weeks after treatment. The SVR12 achieved by both groups were 94% and 78.7% respectively. Moreover, the response rate among treatment experienced patients was 69.0% comparing to 100.0% in the present study. In fact, the sample size in the later study was 70 times higher than our study cohort and this could explain their higher event rates. While in the prior interferon era SVR was measured 24 weeks (SVR24) after the end of treatment, data from clinical trials demonstrated that SVR measured at 12 weeks post-treatment (SVR12) is highly concordant with SVR24 [1, 28].

EVR was a strong predictor of treatment response [29]. In the present study, treatment response at 12 weeks was equivalent to that at 24 weeks. This supports recommendations from phase II and phase III study that SOF+RBV for either 12 or 24 weeks is successful in treating treatment-naïve and treatment-experienced Egyptian patients with genotype 4 HCV although 24 weeks regimen was found more effective than 12 weeks [24–26, 30, 31]. Thus, shorter treatment durations may be possible. However, we do not know if late relapse could occur after that as patients achieving SVR are not followed up after achieving SVR.

Long-term follow-up of chronic HCV patients after completion of antiviral therapy showed that attaining an SVR24 has favorable outcome and that almost 100.0% remain HCV RNA negative years after therapy [30, 32, 33]. The largest studies have shown a minimal relapse rate, between 0 to 1% [34]. Thus, undetectable HCV RNA 24 week after antiviral therapy can be considered a virologic cure. In the real life study conducted by Elsharkawy et al., in Egypt, 50.0% of patients were followed for 12 weeks post-treatment and no data were reported on SVR24. In fact, it is not possible to estimate the treatment success rate in patients who were not followed up. In the present study, the relapse rate was 2.9% and occurred during the second follow up phase, three months following the SVR12. This warrant posttreatment adherent patient monitoring to be extended for at least 6 months to 1 year to better judge the treatment outcome especially that late relapses beyond SVR48 was reported after initially successful DAA therapy [35]. Sarrazin et al., analyzed data from 11 Phase III clinical trials including 3,004 HCV infected patients who received sofosbuvir based therapy and achieved SVR12. Of the 12 people having had a detectable viral load 24 weeks after treatment, five patients (0.17%) experienced viral relapse by the same virus they had before beginning treatment that most probably persisted in the liver or another part of the body and reemerged in the blood by 24 weeks posttreatment [36]. Simmons and co-workers reported in meta-analysis of 49 studies including 8534 patients that the risk of HCV recurrence rises with longer follow up periods. They found that the 5-year risk of late relapse or re-infection post-SVR was 0.9% in HCV mono-infected patients, 8.1% in HCV mono-infected IV drug users or prisoners, and 21.8% in HIV/HCV co-infected patients [37]. Thus, although sustained viral responses at weeks 12 or 24 following completion of HCV therapy may continue to be considered as a major end-point in clinical trials with anti-HCV drugs, it seems worth to advice repeated HCV-RNA testing beyond week 24 postreatment in clinical practice.

The present study is limited by lack of determination of HCV genotype among the study cohort. However, HCV infection in Egypt is predominantly with genotype 4 [6–9]. In fact, HCV genotype was a crucial predictor of SVR in the prior interferon era [38, 39]. The current DAAs combinations are of high efficacy and pan genotypic coverage thus decreasing the role of HCV genotype in predicting treatment response. The 2016 Guidelines provide recommendations on the preferred and alternative DAA regimens by HCV genotype [40]. Therefore, knowing patient genotype is still important for determining the most appropriate treatment regimen. In our study, the overall response rate was 97%. The 3% non-response rate could be attributed is some part to the viral genotype that could be genotype 3, the most difficult to treat. Further studies are warranted to investigate the reasons of non-response. Genotype determination, however, is expensive and not available in all settings. Where genotype information is unavailable, pragmatic decision-making may be required, taking into account the common genotypes circulating in the affected population. However, this advice would only be practicable in countries such as Egypt or Mongolia, where almost all persons are infected with a single genotype [40].

A history of prior HCV treatment was found in previous studies to be significantly related to lack of response with peginterferon based therapy [41, 42] although this does not appear to have the same impact on treatment response to the DAAs, particularly in non-cirrhotic patients. In the present cohort, all non responders were naïve patients with cirrhosis. Interestingly, all experienced patient achieved SVR despite that almost half of them (53.3%) had cirrhosis but none was classified as difficult to treat. This contradict Elsharkawy et al., as they reported that treatment-naive patients had higher SVR rates than the treatment-experienced patients [27].

Likewise, in peginterferon and ribavirin, a high viral load was associated with a 9–16% lower chance of cure [42]. With the use of DAAs, the baseline HCV RNA appears to have little impact on the probability of achieving an SVR [23]. A very high baseline viral level (> 6 million IU/ml) however has been shown to be associated with low likelihood of SVR with shorter duration of therapy with ledipasvir-sofosbuvir [43]. In the present study, viral load did not significantly correlate with SVR. About 8.8% of the study patients had a very high baseline viral level (6–28 million IU/ml).

History of splenectomy was significantly associated with non response (2/4 patients with prior splenectomy) although it did not appear among predictors of non response in our multivariate logistic regression model. Splenectomy was found to improve blood picture and liver function, alleviate portal hypertension and to enhance liver regeneration in patients with liver cirrhosis [44–46]. Evidence from several studies supports that splenectomy prior to interferon-based therapy is safe, alleviates cytopenia and improves the efficacy and adherence to antiviral therapy in cirrhotic patients with portal hypertension and hypersplenism [46–52]. On the other hand, Parana et al., suggested that splenectomy is a negative predictor of response to antiviral therapy in chronic HCV particularly for genotype 1 [51, 53].

Advanced fibrosis and liver cirrhosis is associated with lower cure rates. Severely deteriorated liver condition hampers viral eradication and pledges the occurrence of adverse events [38, 39]. This has persisted to a lesser extent with the use of the DAAs [54, 55]. Liver cirrhosis evidenced by portal hypertension and as reported in other studies [2, 3, 24, 25, 27, 56] was a predictor of nonresponse in our study population.

HCV patients with bilharziasis respond poorly to interferon based antiviral therapy [57]. In our study cohort, having bilharziasis co-infection did not influence treatment response. This could be attributable to treating those found seropositive for bilharziasis with antibilharzial treatment before initiating antiviral therapy.

Although male gender and old ages had low response rates to interferon based therapy, patient demographics do not significantly influence treatment response to DAAs. This mirrors our study. However, male gender was reported by Elsharkawy et al., as a predictor of SVR in chronic HCV Egyptian patients treated with Sofobuvir based regimens [27].

Despite that EVR appeared to be of limited value as a prognostic marker of treatment response in prior DAA-based studies [2, 58], EVR was a strong predictor of treatment response in the present real-life experience. In line with our study, Steinebrunner et al., found in a real-life cohort, that EVR after 4 weeks of treatment was a significant predictor of treatment response in genotype 1 patients [29].

Glucose abnormalities including diabetes mellitus and insulin resistance are strongly associated with HCV infection and have a negative impact on the HCV treatment outcomes [59, 60]. In the present study, prediabetics were less likely to respond to treatment comparing to diabetics suggesting insulin resistance as main player [59, 60]. In fact, diabetics achieved glycemic control under control of antidiabetic therapy that was found to improve the response to antiviral treatment [61]. On the other hand, some studies suggest that the presence of diabetes does not appear to be impact treatment response in genotype 1 HCV patients [62, 63]. High insulin in insulin resistance may inhibit the ability of interferon alfa to block HCV replication due to the activation of PI3K by insulin, thus leading to inhibition of STAT-1, which is involved in the interferon alfa pathway [64]. The impact of diabetes mellitus and insulin resistance on HCV infection and treatment response needs to be further evaluated with the new DAAs.

The main limitation of this study is its sample size, which makes subgroup comparisons difficult.

In conclusion, sofosbuvor is a milestone of a potent treatment regimen with a favorable safety profile for chronic HCV patients infected with genotype 4. sofosbuvir-based therapies resulted in higher SVR rates compared with the previous standard of care. Novel DAAs based therapy should be used to have better response rates especially in patients with advanced fibrosis.

## Supporting information

S1 File SPSS complete HCV study.sav. All data required to replicate the study are available is the supporting information file; “SPSS complete HCV study.sav”.
(SAV)
